# Supplementary material for: Content-rich biological network constructed by mining PubMed abstracts
Source: BMC Bioinformatics. 2004 Oct 8;5:147. doi: 10.1186/1471-2105-5-147 (PMC528731; doi:10.1186/1471-2105-5-147)
Supplement: Additional File 2 — The original results of the above study (non-essential files are deleted to keep the file size under the limit set by BMC bioinformatics). [file 1471-2105-5-147-S2.bz2 › chilibotAdditionalFile2/dip50/335ID8171323E3971/html/TNF_LTA.html]

 


 **TNF** and **LTA** 
  
Found 1446 abstracts in PubMed, retrieved 50.  
 

 What does Google say? 
 PDF only 
| .edu only 

---

**Interactive relationship** (e.g. stimulation, inhibition, etc)

**Neutral relationship**- In contrast, purified  **LTA**  triggered  **TNF**  release at 1 micro g ml.  Ref: 12819051 Infect Immun, 2003
- Effect of  **TNF**  and  **LTA**  polymorphisms on biological markers of response to oxidative stimuli in coal miners a model of gene environment interaction.  Ref: 12566517 J Med Genet, 2003
- Almost all individuals homozygous for the  **TNF**  B2 allele of the  **TNF**  beta  [ **LTA** ]  I polymorphism were also  **TNF**  alpha  [ **TNF** ]  308 G homozygotes.  Ref: 12545017 Crit Care Med, 2003
- Analysis of the maturation process of dendritic cells deficient for  **TNF**  and lymphotoxin alpha  [ **LTA** ]  reveals an essential role for  **TNF** .  Ref: 12885938 J Leukoc Biol, 2003
- The  **TNF**  beta  [ **LTA** ]  I genotype  **TNF**  B1  **TNF**  B2 was associated with significantly higher  **TNF**  alpha  [ **TNF** ]  concentrations than the genotype  **TNF**  B2  **TNF**  B2.  Ref: 12545017 Crit Care Med, 2003
- It binds two types of tumor necrosis factor  [ **TNF** ]  family cytokines, heterotrimer LT alpha  [ **LTA** ]  1 beta 2 and homotrimer LIGHT, and activates multiple signaling pathways including transcriptional factor NF kappa B, c Jun N terminal kinase, and cell death.  Ref: 12571250 J Biol Chem, 2003
- , Malvern, PA, USA, a chimaeric mAb, binds to soluble and membrane bound  **TNF**  alpha  [ **TNF** ] , but not to  **TNF**  beta  [ **LTA** ] .  Ref: 12718738 Expert Opin Biol Ther, 2003
- LPS TLR4 adapted human THP 1 promonocytic cells cross adapt to lipoteichoic acid  **LTA**  TLR2 induced IL 1beta  **TNF**  alpha  [ **TNF** ]  production, suggesting disruption of a common intracellular signaling event s.  Ref: 12691617 J Endotoxin Res, 2003
- LT consists of soluble LT alpha  [ **LTA** ]  3, which binds to  **TNF**  R1 and  **TNF**  R2, and membrane LT alpha  [ **LTA** ]  1 beta 2, which binds to LT beta R.  Ref: 12882833 Int Immunol, 2003
- The production of  **TNF**  alpha  [ **TNF** ]  and IFN gamma was similar in the  **LTA**  and control groups, and both were higher than the levels in the RP group.  Ref: 12653844 Clin Exp Immunol, 2003
- Application of the approach to the  **TNF**   **LTA**  locus identified functionally important haplotypes that correlate with allele specific transcription of  **LTA** .  Ref: 12627232 Nat Genet, 2003
- CONCLUSION The AA genotype at the lymphotoxin alpha  [ **LTA** ]  250 site is associated with higher serum tumor necrosis factor alpha  [ **TNF** ]  levels and a higher mortality in children with bacteremia.  Ref: 12780951 Pediatr Crit Care Med< MedlineTA>Pediatr Crit Care Med, 2003
- Samples homozygous at TNFA  [ **TNF** ]  308 and BAT1 intron 10 demonstrated identity between the African TNFA  [ **TNF** ]  308\*2 haplotype, the 8.1AH and the Asian diabetogenic 58.1AH HLA A33, B58, DR3 across a region spanning BAT1, ATP6G, IKBL,  **LTA** , TNFA  [ **TNF** ] , LTB, LST 1 and AIF 1.  Ref: 12859597 Tissue Antigens, 2003
- Serum tumor necrosis factor alpha  [ **TNF** ]  levels were 324 124 pg mL in bacteremic children with the AA genotype at the lymphotoxin alpha  [ **LTA** ]  250 site compared with 92 59 pg mL in bacteremic children with the AG genotype p < ;.  Ref: 12780951 Pediatr Crit Care Med< MedlineTA>Pediatr Crit Care Med, 2003
- Two polymorphic nucleotides in the  **TNF**  cluster G 308A  **TNF**  alpha  [ **TNF** ]  and G 252A  **TNF**  beta  [ **LTA** ] , associated with increased  **TNF**  alpha  [ **TNF** ]  production, were shown to be in tight linkage disequilibrium with the class I and II HLA loci, generating HLA haplotypes with extended linkage disequilibrium.  Ref: 12714268 Mech Ageing Dev, 2003
- Genetic polymorphisms located in the first intron of the lymphotoxin alpha  [ **LTA** ]  gene have been associated with increased secretion of tumor necrosis factor alpha  [ **TNF** ] .  Ref: 12780951 Pediatr Crit Care Med< MedlineTA>Pediatr Crit Care Med, 2003
- Strikingly, such  **LTA**  adapted cells still respond to LPS stimulation of TLR4 with rapid activation and degradation of IRAK1, and robust IL 1beta  **TNF**  alpha  [ **TNF** ]  production.  Ref: 12691617 J Endotoxin Res, 2003

**Non-interactive relationship** (e.g. studied together, co-existance, homology, etc.)

- To evaluate the association of  **TNF**  alpha  [ **TNF** ]  TNFA  [ **TNF** ]  and  **TNF**  beta  [ **LTA** ]  TNFB  [ **LTA** ]  polymorphisms with psoriasis in the Korean population, we investigated  **TNF**  alpha  [ **TNF** ]  238 and 308 promoter region and  **TNF**  beta  [ **LTA** ]  NcoI polymorphism using PCR RFLP in 103 Korean psoriasis patients and 125 normal controls.  Ref: 12709814 Arch Dermatol Res, 2003
- In contrast, mice deficient in both tumor necrosis factor  [ **TNF** ]  and lymphotoxin alpha  [ **LTA** ]  TNFalpha x LTalpha or in lymphocytes RAG 1, micro MT, in which numbers of PPs are reduced in number, were highly resistant to oral challenge, and their intestines were virtually devoid of prion infectivity at all times after challenge.  Ref: 12651603 Am J Pathol, 2003
- We examined polymorphisms in the genes for tumor necrosis factor  [ **TNF** ]   **TNF** ,  **TNF**  receptor type 1 and 2, lymphotoxin LT alpha  [ **LTA** ] , interleukin IL 1beta, IL 6, IL 10, monocyte chemoattractant protein MCP 1, and mannose binding protein ManBP in 143 HTLV I carriers whether these polymorphisms affect the provirus load in the PBMCs of carriers.  Ref: 12651071 Hum Immunol, 2003
- Linkage disequilibrium between tumor necrosis factor  [ **TNF** ]   **TNF**  alpha  [ **TNF** ]  308 G A promoter and  **TNF**  beta  [ **LTA** ]  NcoI polymorphisms Association with  **TNF**  alpha  [ **TNF** ]  response of granulocytes to endotoxin stimulation.  Ref: 12545017 Crit Care Med, 2003
- The aim of this investigation was to determine whether a determinant in the first intron of the  **TNF**  beta  [ **LTA** ]  gene  **TNF**  beta  [ **LTA** ]  252 and two promoter region polymorphisms of the  **TNF**  alpha  [ **TNF** ]  gene  **TNF**  alpha  [ **TNF** ]  308 and  **TNF**  alpha  [ **TNF** ]  238 affect susceptibility to multiple sclerosis MS.  Ref: 12568117 Autoimmunity, 2002
- The blood samples were taken for measurement of IL 1,  **TNF**  alpha  [ **TNF** ] , IL 6 and IL 10,  **TNF**  beta  [ **LTA** ] .  Ref: 12575195 Hua Xi Yi Ke Da Xue Xue Bao, 2002
- Immunohistochemical and RT PCR analyses have shown elevated expression of pro inflammatory IL 6,  **TNF**  beta  [ **LTA** ] , ICAM 1 and pro fibrogenic TGF beta cytokines, and BMP 7 treatment significantly reduced their expression in the intestine.  Ref: 12811818 J Cell Physiol, 2003
- Polymorphisms in the 252 A G lymphotoxin alpha  [ **LTA** ]  and the 308 A G tumor necrosis factor alpha  [ **TNF** ]  genes and susceptibility to chronic periodontitis in a Czech population.  Ref: 12828656 J Periodontal Res, 2003
- The risk of prolonged mechanical ventilation at 4, and 48 hrs was higher for patients with a GA or GG genotype at the LT alpha  [ **LTA** ]  250 site and the LT alpha  [ **LTA** ]  250G  **TNF**  308G haplotype.  Ref: 12545006 Crit Care Med, 2003
- mRNA was reverse transcribed into single strand cDNA and then used as a probe hybridizing to the DNA fragments of col I alpha, col I alpha, col III, col V, fibronectin, IL 1, IL 6, TGF beta, LIF,  **TNF**  alpha  [ **TNF** ] ,  **TNF**  beta  [ **LTA** ]  by reverse northern and dot blot hybrization.  Ref: 12548865 Shi Yan Sheng Wu Xue Bao, 1999
- 0001 linkage disequilibrium was found for the  **TNF**  beta  [ **LTA** ]  I and the  **TNF**  alpha  [ **TNF** ]  308 genetic polymorphisms.  Ref: 12545017 Crit Care Med, 2003
- Implications of serum  **TNF**  beta  [ **LTA** ]  and IL 13 in the treatment response of childhood nephrotic syndrome.  Ref: 12697154 Cytokine, 2003
- No significant differences were found in the genotype distribution of  **TNF**  alpha  [ **TNF** ]  308 and TNFB  [ **LTA** ]  among different groups.  Ref: 12679941 World J Gastroenterol, 2003
- Independent of linkage disequilibrium with class II HLA,  **TNF**  haplotype TNFA  [ **TNF** ]  307\*2 TNFB  [ **LTA** ]  252\*2 showed a significant protective effect OR = 0.031, partly exacerbated by partial linkage to protective class II haplotypes.  Ref: 12622777 Tissue Antigens, 2003
- **TNF**  alpha  [ **TNF** ]  and  **TNF**  beta  [ **LTA** ]  induced a 3 to 24 fold increase in IL 8 protein expression of BEC, and a 2 to 8 fold increased IL 8 expression in estrogen independent BCC cell lines and no significant IL 8 expression in estrogen dependent cell lines.  Ref: 12792762 Oncol Rep< MedlineTA>Oncol Rep, 2003
- TNFA  [ **TNF** ]  TNFB  [ **LTA** ]  haplotypes modify susceptibility to type I diabetes mellitus independently of HLA class II in a Moroccan population.  Ref: 12622777 Tissue Antigens, 2003
- At least two polymorphisms seem to have functional relevance in the respective genes TNFA  [ **TNF** ]  307 and TNFB  [ **LTA** ]  252.  Ref: 12622777 Tissue Antigens, 2003
- DA persisted and induced inflammatory demyelination in LT alpha  [ **LTA** ]  but not  **TNF**  weanling mice of a normally resistant haplotype C57BL 6, suggesting that LT, but not  **TNF** , is critical for resistance to DA infection.  Ref: 12882833 Int Immunol, 2003
- Members of the tumour necrosis factor superfamily TNFSF  [ **LTA** ]  and  **TNF**  receptor superfamily TNFRSF have crucial roles in both innate and adaptive immunity.  Ref: 12860530 Trends Immunol, 2003
- Miners were genotyped for common functional polymorphisms in the gene for tumour necrosis factor alpha  **TNF**  and lymphotoxin alpha  [ **LTA** ]   **LTA** , two proinflammatory cytokines that have been implicated in the pathogenesis of chronic lung diseases.  Ref: 12566517 J Med Genet, 2003
- **TNF**  alpha  [ **TNF** ]  and beta gene polymorphisms in multiple sclerosis a highly significant role for determinants in the first intron of the  **TNF**  beta  [ **LTA** ]  gene.  Ref: 12568117 Autoimmunity, 2002
- We investigated the association of polymorphisms in the tumor necrosis factor  [ **TNF** ]   **TNF**  microsatellite as well as the promoter region of the TNFA  [ **TNF** ]  gene and the TNFB  [ **LTA** ]  gene with palmoplantar pustulosis PPP.  Ref: 12691703 Hum Immunol, 2003
- This study demonstrates the significance of serum  **TNF**  beta  [ **LTA** ]  and IL 13 levels in relation to the disease activity and treatment response of childhood NS.  Ref: 12697154 Cytokine, 2003
- Patients with acute NS had higher serum  **TNF**  beta  [ **LTA** ]  and IL 13 levels than normal controls.  Ref: 12697154 Cytokine, 2003
- Because it contains many genes related to inflammatory and immune responses, including tumor necrosis factor  [ **TNF** ]   **TNF** , lymphotoxin alpha  [ **LTA** ]  LT alpha  [ **LTA** ] , and allograft inflammatory factor 1 AIF 1 genes, it is unclear which gene within the class III region is responsible for the susceptibility to the disease.  Ref: 12559634 Hum Immunol, 2003
- ... the levels of IL 11 and LIF were decreased with increasing amounts of IL 13, but were increased with increasing amounts of  **TNF**  beta  [ **LTA** ] .  Ref: 12620497 Fertil Steril, 2003
- When media conditioned by Lactobacillus rhamnosus GG LGG are co incubated with lipopolysaccharide LPS or lipoteichoic acid  **LTA** ,  **TNF**  alpha  [ **TNF** ]  production is significantly inhibited compared to controls.  Ref: 12675685 Cell Microbiol, 2003
- Thus, an altered Th1 Th2 reaction as demonstrated by  **TNF**  beta  [ **LTA** ]  IL 13 imbalance may play a pathophysiologic role in childhood NS.  Ref: 12697154 Cytokine, 2003
- OBJECTIVE To evaluate the effects of interleukin IL 13, a T helper Th 2 cytokine, and tumor necrosis factor  [ **TNF** ]   **TNF**  beta  [ **LTA** ] , a Th1 cytokine, on the production of IL 6 family cytokines and chemokines by endometrial stromal cells ESC.  Ref: 12620497 Fertil Steril, 2003
- We studied the biallelic interleukin 10 1082 promoter, the tumor necrosis factor alpha  [ **TNF** ]  308 promoter and the lymphotoxin alpha  [ **LTA** ]  polymorphisms with regard to the development of septic shock in pneumococcal infection.  Ref: 12746253 Am J Respir Crit Care Med< MedlineTA>Am J Respir Crit Care Med, 2003
- This study was conducted to test the association between the tumor necrosis factor  [ **TNF** ]   **TNF**  beta  [ **LTA** ]  gene B polymorphism and schizophrenia in the Korean population.  Ref: 12648734 Schizophr Res, 2003
- **TNF**  beta  [ **LTA** ]  and  **TNF**  alpha  [ **TNF** ]  308 polymorphisms were performed by analyzing NcoI digested DNA fragment obtained by polymerase chain reaction PCR.  Ref: 12888330 J Surg Res, 2003
- CONCLUSION S These results suggest that IL 13 and  **TNF**  beta  [ **LTA** ]  secreted in the cyclic endometrial tissue and in the decidua may differentially regulate the production of IL 6 family cytokines and chemokines by ESC.  Ref: 12620497 Fertil Steril, 2003
- **TNF**  beta  [ **LTA** ]  252 genotypes were significantly associated with MS The frequency of  **TNF**  beta  [ **LTA** ]  was increased p = 0.00009 while the frequency of  **TNF**  beta  [ **LTA** ]  was decreased p = 0.0012 in MS patients as compared to controls.  Ref: 12568117 Autoimmunity, 2002
- METHODS In this study, we investigated an association between chronic periodontitis and two previously described bi allelic polymorphisms in the  **TNF**  locus a G to A transition at position 308 in the 5 promoter region of the  **TNF**  alpha  [ **TNF** ]  gene and an NcoI restriction fragment length polymorphism RFLP in the first intron position 252A G of the lymphotoxin alpha  [ **LTA** ]  LT alpha  [ **LTA** ]  gene.  Ref: 12828656 J Periodontal Res, 2003
- Both Lymphotoxin alpha  [ **LTA** ]  and  **TNF**  Are Crucial for Control of Toxoplasma gondii in the Central Nervous System.  Ref: 12794148 J Immunol< MedlineTA>J Immunol, 2003
- After 4 weeks of prednisolone treatment, patients with steroid resistant NS SRNS presented a higher serum  **TNF**  beta  [ **LTA** ]  level than that before treatment p=0.008.  Ref: 12697154 Cytokine, 2003
- The following polymorphisms were investigated  **TNF**  308, the biallelic polymorphism located in the first intron of the lymphotoxin alpha  [ **LTA** ]  gene, and exon 1 and exon 6 of the  **TNF**  receptor 1 and 2 genes, respectively.  Ref: 12661999 Eur Respir J, 2003
- RESULT S The increase in levels of IL 6, IL 8, MCP 1, and eotaxin in the culture media of ESC paralleled the addition of increasing amounts of IL 13 and  **TNF**  beta  [ **LTA** ] .  Ref: 12620497 Fertil Steril, 2003
- In the present study, we measured the Th1 Th2 cytokines, serum tumor necrosis factor  [ **TNF** ]  beta  **TNF**  beta  [ **LTA** ]  and interleukin 13 IL 13, levels in children with NS before and after prednisolone 60mg m 2 day treatment for 4 weeks, to evaluate their relationships with disease activity and treatment response.  Ref: 12697154 Cytokine, 2003
- Moreover, cells adapted to either  **LTA**  or LPS production of IL 1beta  **TNF**  alpha  [ **TNF** ]  normally produce soluble interleukin 1 receptor antagonist sIL 1Ra anti inflammatory protein when stimulated by either agonist.  Ref: 12691617 J Endotoxin Res, 2003
- Patients with SRNS appeared to have elevated  **TNF**  beta  [ **LTA** ]  after steroid therapy, while patients with SSNS tended to have elevated IL 13 after steroid therapy.  Ref: 12697154 Cytokine, 2003
- This activity of LT depends on membrane LT alpha  [ **LTA** ]  1 beta 2 and not soluble LT alpha  [ **LTA** ]  3, since DA virus persisted and induced inflammatory demyelination in LT beta R, but not  **TNF**  R1 or  **TNF**  R2, mice.  Ref: 12882833 Int Immunol, 2003
- However, the significant differences in the frequencies of the combined genotypes  **TNF**  alpha  [ **TNF** ]  and LT alpha  [ **LTA** ]  between the control and the patient groups were found using a simulation by applying the Monte Carlo method P <.  Ref: 12828656 J Periodontal Res, 2003
- LC 46, an HBx 52 60 specific clone, is CD62L CD69 CD45RO CD45RA CD25 dim and is stained by IFN gamma approximately 92%, IL 2 30%, and  **TNF**  alpha  [ **TNF** ]  56%, but not by IL 5, IL 10, IL 12, or  **TNF**  beta  [ **LTA** ] , indicating that the cells are fully activated T cytotoxic 1 type cells.  Ref: 12538674 J Immunol, 2003
- Tumor necrosis factor  [ **TNF** ]   **TNF**  alpha  [ **TNF** ]  and  **TNF**  beta  [ **LTA** ]  are proinflammatory cytokines that have been implicated in the pathogenesis of several autoimmune diseases.  Ref: 12568117 Autoimmunity, 2002
- CONCLUSIONS The LT alpha  [ **LTA** ]  250 and LT alpha  [ **LTA** ]  250G  **TNF**  308G haplotypes are associated with prolonged mechanical ventilation after coronary artery bypass graft.  Ref: 12545006 Crit Care Med, 2003
- INTERVENTION S The ESC were incubated for 24 hours with recombinant human IL 13 and recombinant human  **TNF**  beta  [ **LTA** ] .  Ref: 12620497 Fertil Steril, 2003
- The different expression patterns of the two distinct TNFR homologues may be critical in determining whether binding with  **TNF**  alpha  [ **TNF** ]  or  **TNF**  beta  [ **LTA** ]  have activating, proliferative, or apoptotic effects on target cells.  Ref: 12631519 Dev Comp Immunol, 2003
- CONCLUSION Our data suggest that combined genotypes composed of the  **TNF**  alpha  [ **TNF** ]  and LT alpha  [ **LTA** ]  gene polymorphisms may influence the susceptibility to chronic periodontitis.  Ref: 12828656 J Periodontal Res, 2003
- Although there were significant differences between type 1 diabetes patients and controls in the distributions of  **TNF**  promoter polymorphisms at position 1031 and 857, and LT alpha  [ **LTA** ]  gene NcoI polymorphism, none of them was independently associated with the disease after two locus analysis with HLA class II alleles.  Ref: 12559634 Hum Immunol, 2003
- NcoI polymorphism exists in  **TNF**  gene promoter and influences  **TNF**  alpha  [ **TNF** ]  gene transcription.AIMTo determine the predictive value of plasma levels of  **TNF**  alpha  [ **TNF** ]  and its soluble receptors sTNFR,  **TNF**  alpha  [ **TNF** ]  308 and TNFB  [ **LTA** ]  polymorphisms on the occurrence of ASP and ASP associated early septic shock.METHODOLOGYGenotypes were determined in patients n = 208 and healthy controls n = 116 by means of restriction fragment length polymorphism analysis of polymerase chain reaction products.  Ref: 12717265 Pancreas, 2003
- Expression of cytokines  **TNF**  beta  [ **LTA** ]  and LT beta preceded, and the expression of TGF beta1 followed chemokine upregulation.  Ref: 12828562 Scand J Immunol, 2003
- Immunity to Toxoplasma gondii critically depends on TNFR type I mediated immune reactions, but the precise role of the individual ligands of TNFR1,  **TNF**  and lymphotoxin alpha  [ **LTA** ]  LTalpha, is still unknown.  Ref: 12794148 J Immunol< MedlineTA>J Immunol, 2003
- Neutralizing antibodies against human RANTES, MIP 1alpha, MIP 1beta, alpha interferon IFN alpha, IFN beta, IFN gamma, interleukin 4 IL 4, IL 10, IL 13, IL 16, MCP 1, MCP 3, tumor necrosis factor alpha  [ **TNF** ]   **TNF**  alpha  [ **TNF** ] , or  **TNF**  beta  [ **LTA** ]  failed to reverse the HIV 1 suppressive activity.  Ref: 12885891 J VirolJ Virol, 2003
- AIM To investigate  **TNF**  alpha  [ **TNF** ]  308 and TNFB  [ **LTA** ]  polymorphisms in acute biliary pancreatitis ABP and to related them to the plasma  **TNF**  alpha  [ **TNF** ]  levels.  Ref: 12679941 World J Gastroenterol, 2003
- To analyze the involvement of tumor necrosis factor  [ **TNF** ]   **TNF**  and the related cytokine lymphotoxin LT alpha  [ **LTA** ]  in DC maturation, we studied the development of DCs from the BM of B6. **TNF** , B6.LTalpha, and B6. **TNF**  LTalpha mice and compared it to B6.WT mice.  Ref: 12885938 J Leukoc Biol, 2003
- We attempted to study the effect of polymorphisms at the 308 site in the promoter region of the tumor necrosis factor  [ **TNF** ]  gene  **TNF**  308 and the 250 site within the lymphotoxin alpha  [ **LTA** ]  gene LT alpha  [ **LTA** ]  250 on the risk of prolonged mechanical ventilation after coronary artery bypass grafting.  Ref: 12545006 Crit Care Med, 2003
- These results suggest that the  **TNF**  beta  [ **LTA** ]  gene plays a significant role in the etiopathogenesis of MS.  Ref: 12568117 Autoimmunity, 2002
- HI induced an increase in mRNA expression for IL 1beta,  **TNF**  alpha  [ **TNF** ]  and  **TNF**  beta  [ **LTA** ] , but there was no difference between the ipsi and contralateral hemispheres.  Ref: 12640177 Dev Neurosci, 2002
- DESIGN The effects of IL 13 and  **TNF**  beta  [ **LTA** ] , on the production of IL 6, IL 11, leukemia inhibitory factor LIF, IL 8, growth regulated oncogene alpha GROalpha, monocyte chemoattractant protein 1 MCP 1, regulated on activation, T cell expressed and secreted RANTES, and eotaxin were investigated.  Ref: 12620497 Fertil Steril, 2003
- Then, we investigated AIF 1,  **TNF** , and LT alpha  [ **LTA** ]  gene polymorphisms in 165 patients with type 1 diabetes, consisting of 90 patients with young onset type 1 diabetes, 75 patients with adult onset type 1 diabetes, and 200 control patients.  Ref: 12559634 Hum Immunol, 2003
